# Determining the Binding Affinity of Therapeutic Monoclonal Antibodies towards Their Native Unpurified Antigens in Human Serum

**DOI:** 10.1371/journal.pone.0080501

**Published:** 2013-11-06

**Authors:** Christine Bee, Yasmina N. Abdiche, Jaume Pons, Arvind Rajpal

**Affiliations:** Rinat, Pfizer Inc., South San Francisco, California, United States of America; Cordelier Research Center, INSERMU872-Team16, France

## Abstract

Monoclonal antibodies (mAbs) are a growing segment of therapeutics, yet their *in vitro* characterization remains challenging. While it is essential that a therapeutic mAb recognizes the native, physiologically occurring epitope, the generation and selection of mAbs often rely on the use of purified recombinant versions of the antigen that may display non-native epitopes. Here, we present a method to measure both, the binding affinity of a therapeutic mAb towards its native unpurified antigen in human serum, and the antigen’s endogenous concentration, by combining the kinetic exclusion assay and Biacore’s calibration free concentration analysis. To illustrate the broad utility of our method, we studied a panel of mAbs raised against three disparate soluble antigens that are abundant in the serum of healthy donors: proprotein convertase subtilisin/kexin type 9 (PCSK9), progranulin (PGRN), and fatty acid binding protein (FABP4). We also determined the affinity of each mAb towards its purified recombinant antigen and assessed whether the interactions were pH-dependent. Of the six mAbs studied, three did not appear to discriminate between the serum and recombinant forms of the antigen; one mAb bound serum antigen with a higher affinity than recombinant antigen; and two mAbs displayed a different affinity for serum antigen that could be explained by a pH-dependent interaction. Our results highlight the importance of taking pH into account when measuring the affinities of mAbs towards their serum antigens, since the pH of serum samples becomes increasingly alkaline upon aerobic handling.

## Introduction

Therapeutic monoclonal antibodies (mAbs) are an expanding segment of the drug market, especially in the quest for novel drugs to treat cancer and chronic diseases. While generating a mAb with an optimum affinity for the target antigen is an important factor in achieving an efficacious and safe therapeutic agent, the biophysical *in vitro* characterization of antigen/mAb interactions under physiologically relevant conditions can be challenging. Only mAbs that recognize native epitopes can be considered as therapeutic candidates, however, many steps in the mAb discovery process rely on the use of purified antigens, which are often from recombinant sources and may present non-native epitopes. Label-free surface plasmon resonance (SPR)-based biosensors, such as Biacore, are commonly employed to determine the affinities of antigen/mAb interactions and typically require the use of purified antigen. While SPR-based measurements often yield affinities that agree well with solution methods [1–4], the use of a surface can sometimes give misleading results due to the introduction of artifacts, which may be influenced by the choice of sensor chip type used [5] and the relative charges of the analyte/ligand pair being studied [6]. The kinetic exclusion assay (KinExA) is a method for determining the solution affinities of protein/protein interactions and it enables the study of unpurified proteins at low concentrations in various buffers [3,7]. Here, we extend the application of the KinExA to the study of mAbs interacting with their native unpurified antigens, as available in human serum. We use it to determine both the apparent affinity of the mAb for its native target and the target’s endogenous concentration in serum. To test our method, we studied three unrelated monomeric model antigens that are abundant in normal human serum, namely proprotein convertase subtilisin/kexin type 9 (PCSK9), progranulin (PGRN), and fatty acid binding protein (FABP4). We studied two mAbs per antigen and for each mAb, we compared its affinity towards its purified recombinant antigen and its unpurified serum antigen. 

## Results

### The KinExA was amenable to studying unpurified native antigens in undiluted human serum

**Figure 1:**
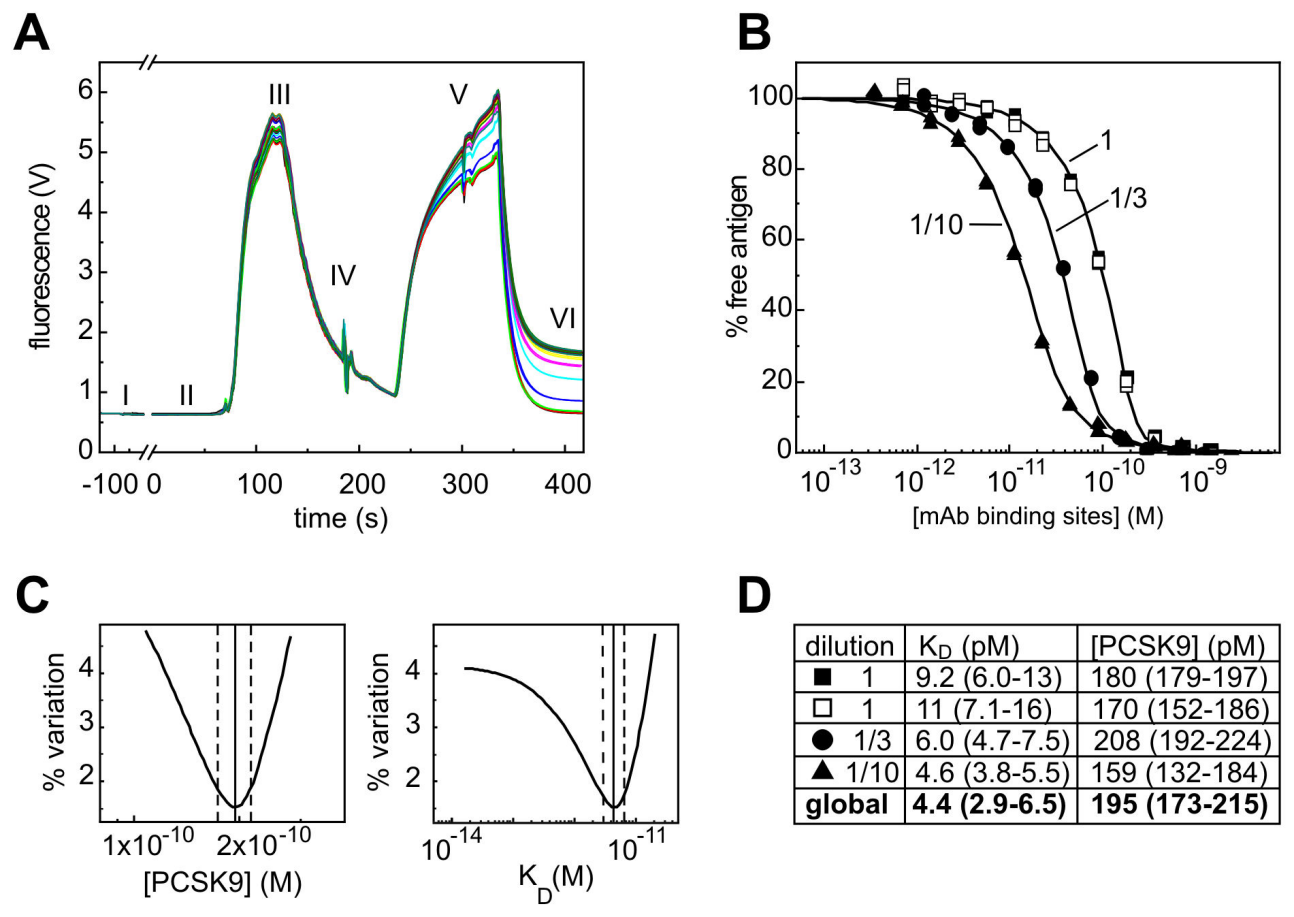
Human serum titrated with anti-PCSK9 mAb J16. (A) Raw data trace of fluorescence (in Volts) as a function of time recorded by the KinExA instrument for a typical experiment: (I) packing of mAb-coated beads inside the flow cell; (II) baseline signal; (III) auto-fluorescence signal obtained from serum components (presumably porphyrins); (IV) buffer wash; (V) detection of bead-captured PCSK9 with a Dylight-labeled mAb; and (VI) buffer wash, after which the final fluorescence signal for bead-captured PCSK9 is recorded (relative to the baseline signal). (B) Global fit of normalized data obtained from titrating J16 into different dilutions of serum prepared in PBS. (C) Error plots for K_D_ and PCSK9 concentration for the global analysis in panel B with best fit values (solid line) and 95% confidence interval (dotted lines). (D) Comparison of the fits obtained for single-curve and multi-curve (global) analysis of the data in panel B. The PCSK9 concentration is back-calculated for undiluted serum. Open and closed symbols indicate independent experiments performed with the same dilution factor.

The KinExA offers two distinct assay formats for determining the apparent affinity of an antigen/mAb interaction and the results are typically independent of the assay format employed [3]. Here, we opted for the “fixed antigen” assay orientation because it is amenable to working with serum, which may contain a low and unknown concentration of the native antigen of interest. Figure 1A shows the raw data trace obtained for a typical experiment where undiluted human serum was titrated with the anti-PCSK9 mAb J16 [8]. In contrast to experiments in standard buffers such as PBS, the autofluorescence of serum allows one to visualize its passage through the flow cell but does not disturb the end point measurement. Figure 1B shows the results of globally fitting curves from independent experiments where J16 was titrated into undiluted serum or serum that was diluted in running buffer. The resulting error plots (Figure 1C) show that the global analysis provided good precision for both the apparent K_D_ value and the apparent PCSK9 concentration contained in human serum. Comparing the fit values obtained for a single-curve analysis versus a multi-curve global analysis (Figure 1D) reveals that a single curve (in this example, the one obtained using a three-fold dilution of the serum) can yield both an apparent K_D_ and an antigen concentration that fall within the 95% confidence interval of the global analysis. We refer to this information-rich curve as a “sweet spot” curve [3].

### Six antigen/mAb interactions with a thousand-fold K_D_ range were studied in serum

**Figure 2:**
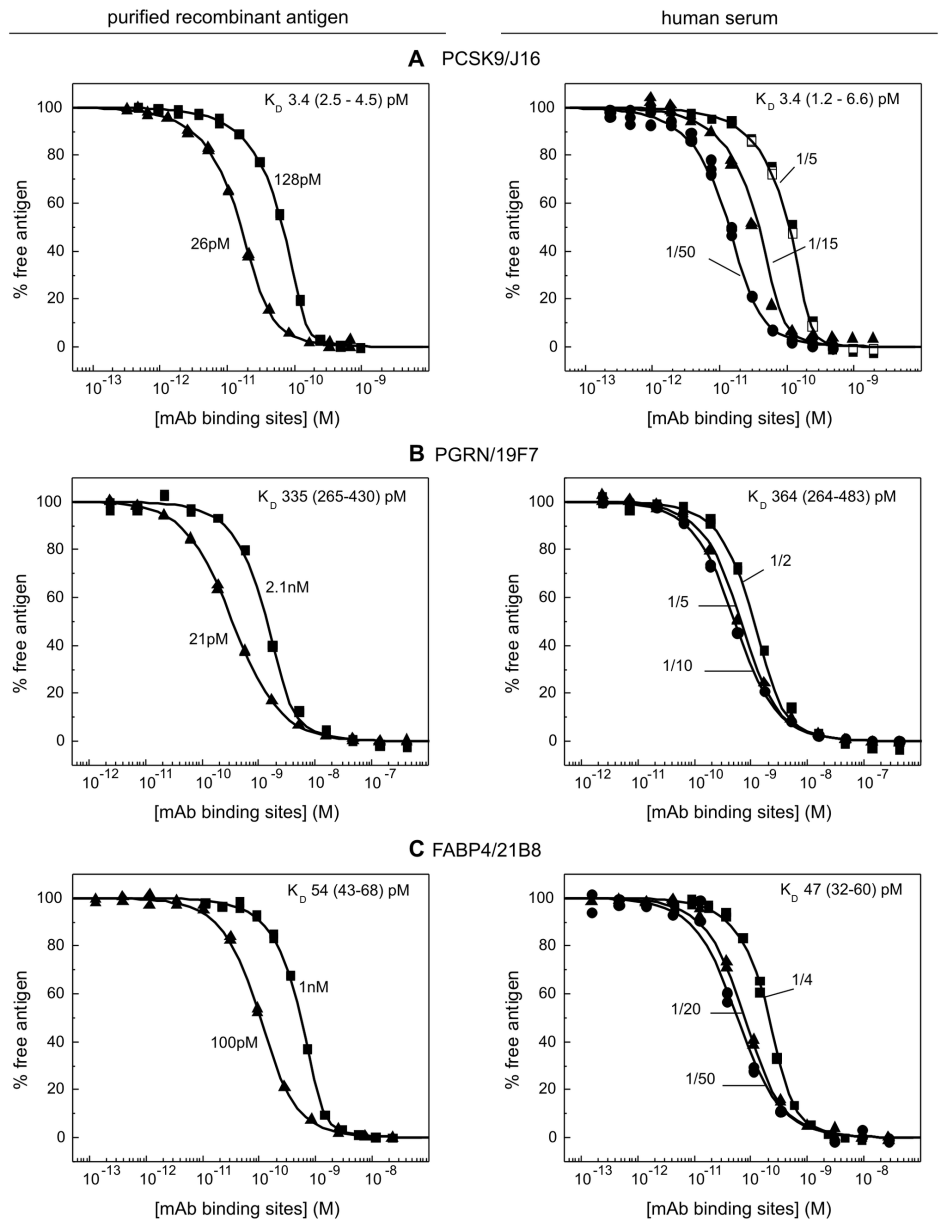
KinExA-determined affinities of three different mAbs (J16, 19F7, and 21B8) towards their purified recombinant antigens (left) and native antigens in human serum (right). The fixed concentration of recombinant antigen (as determined by CFCA) used to generate each titration curve is indicated on the plots. For serum assays, the dilution factor is shown instead. The best fit K_D_ (and 95% confidence interval) is reported on each panel.

**Figure 3:**
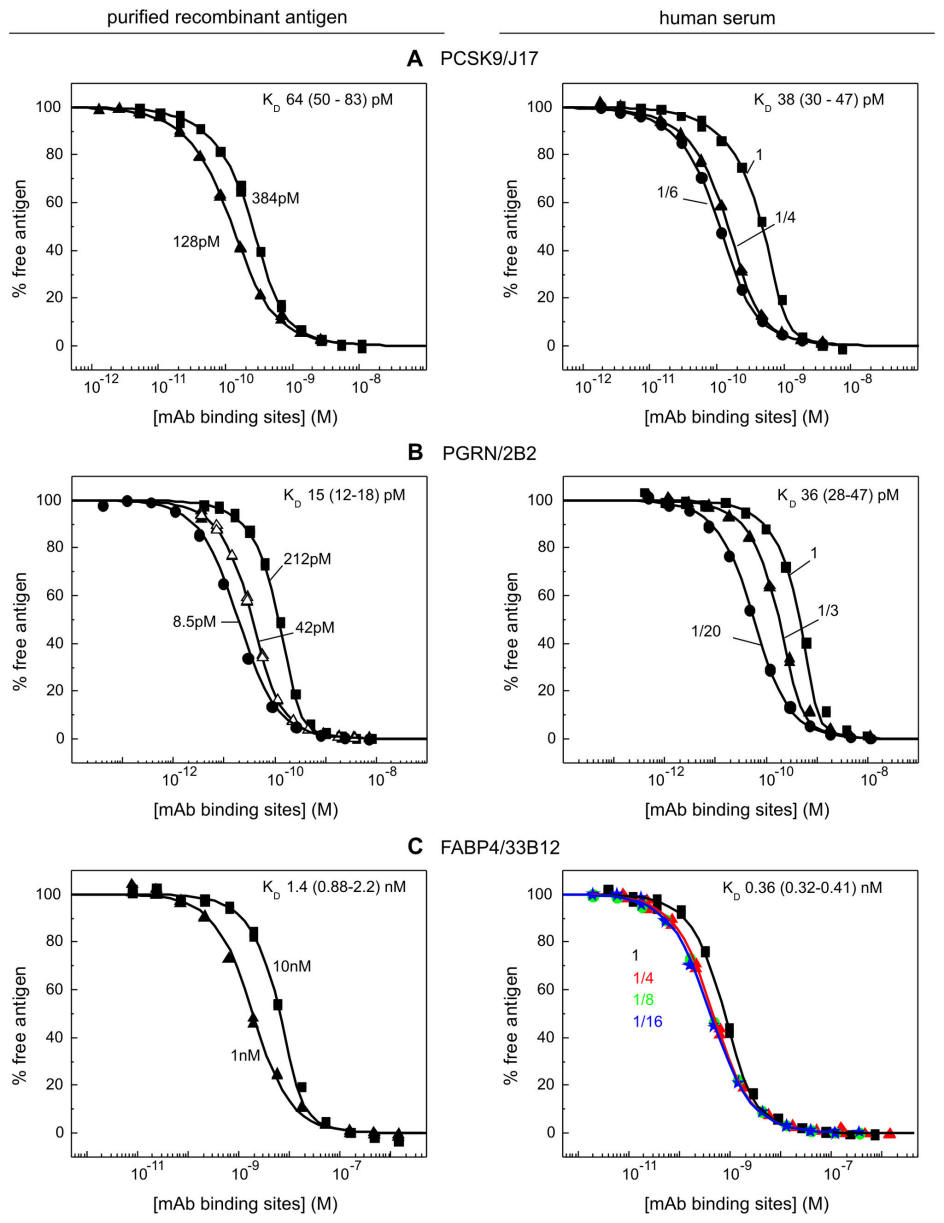
KinExA-determined affinities of three mAbs (J17, 2B2, and 33B12) towards their purified recombinant antigens (left) and native antigens in human serum (right). The data are presented in the same way as in Figure 2.

Figure 2, Figure 3 and Table 1 summarize the affinity data obtained for six mAbs binding their purified recombinant antigens and native unpurified antigens in human serum. For PCSK9, we selected mAbs J16 and J17, which recognize the same epitope but were engineered to have affinities that were influenced differently by pH [8,9]. For PGRN, we chose mAbs 2B2 and 19F7, which bound to different subdomains of PGRN such that each could serve as secondary detection reagent for the other. Similarly, we selected two anti-FABP4 mAbs, 21B8 and 33B12, which bound non-overlapping epitopes. For all interactions studied, the data obtained from a global analysis of independent experiments conducted in various dilutions of human serum were well-described by a bimolecular binding equation, suggesting that there was no detectable perturbation in the apparent K_D_ values when working in undiluted serum compared with highly diluted serum. Our model interactions spanned a thousand-fold K_D_ range, from single digit pM to single digit nM, illustrating the broad dynamic range that is accessible to this method. 

The analysis of a KinExA experiment requires the concentration of one binding partner to be known, which must be the mAb when the purpose of the experiment is to study its interaction in serum, because the antigen’s endogenous concentration is not known *a priori*. We employed Biacore’s calibration-free concentration analysis (CFCA) to determine the active concentrations of our mAbs because, unlike titration-based methods, it does not require a standard compound. However, since the CFCA relied upon immobilizing and regenerating the antigens to provide high enough surface capacities to achieve mass transport-limited binding of the mAb as analyte, it was not amenable to all six mAbs in our panel. To bypass this limitation, we reversed the assay orientation of the CFCA and instead determined the active concentration of our three recombinant antigens, taking advantage of the facile immobilization and regeneration of our mAbs. Using the KinExA, we then titrated our mAbs (unknowns) against their recombinant antigens (knowns). Thus, by entering the antigen’s CFCA-determined active concentration we determined the active concentration of its interacting mAb, by allowing it to be a floated parameter in the analysis. The so-determined active concentration of the mAb was then entered as a known parameter in our serum experiments. While this was an indirect route to determining the active concentrations of our mAbs, we were confident in this approach because, for those mAbs that were amenable to CFCA (J16, J17, and 2B2), we obtained excellent agreement between the active concentrations determined by these two complementary methods (see Table 1). For J16 and J17, we were able to bypass the need for immobilized antigen by using antigen-blocking anti-idiotypic mAbs as surrogates to probe the active binding sites of J16 and J17.

**Table 1:** Comparison of KinExA-determined affinities for various mAbs binding their purified recombinant and native serum antigens.

			**mAb’s activity (%)^a^**		**recombinant antigen**		**human serum**			
**Figure**	**mAb**	**antigen**	**CFCA**	**LCM**	**K_D_ (pM)**	**n**	**K_D_ (pM)**	**antigen conc.^b^ (nM)**	**n**	**K_D_ ratio^c^**
2A	J16	PCSK9	44	47 (45-51)	3.4 (2.5-4.5)	2	3.4 (1.2-6.6)	1.1 (0.85-1.3)	5	1.0
2B	19F7	PGRN	nd	41 (35-51)	335 (265-430)	2	364 (264-483)	1.5 (0.92-2.2)	3	1.1
2C	21B8	FABP4	nd	75 (68-83)	54 (43-68)	2	47 (32-60)	1.3 (0.89-1.8)	3	0.9
3A	J17	PCSK9	57	53 (47-61)	64 (50-83)	2	38 (30-47)	0.88 (0.80-0.98)	3	0.6^*d*^
3B	2B2	PGRN	48	38 (34-43)	15 (12-18)	4	36 (28-47)	0.85 (0.73-0.98)	3	2.4^*d*^
3C	33B12	FABP4	nd	70 (52-97)	1390 (883-2200)	2	360 (317-406)	0.83 (0.54-1.1)	4	0.26

^a^ apparent activity of mAb (expressed as a percent of its nominal concentration) as determined via Biacore’s CFCA or the KinExA’s fitted LCM converted into percent activity (where LCM of 1 = 100%). The 95% confidence interval of the KinExA fit is shown in parentheses.

^b^ in undiluted serum

^c^ K_D_ ratio of serum antigen/recombinant antigen

^d^ the difference in apparent K_D_ values between purified recombinant antigen and serum antigen is pH-influenced

n = the number of independent titration curves incorporated in the global fitnd = not determined

### Three mAbs did not discriminate between the recombinant and native forms of their respective antigens

Figure 2A shows that a dual-curve KinExA analysis of J16’s interaction with purified rhPCSK9 yielded an apparent K_D_ of 3.4 pM with a 95% confidence interval of 2.5 pM to 4.5 pM, which recapitulated our previously published value [8]. J16’s apparent K_D_ for serum PCSK9 was identical to the affinity determined for rhPCSK9. A similar K_D_ value was obtained using an independent serum lot (same supplier, but pooled from different donors) – data from this lot were shown in Figure 1. Anti-PGRN mAb 19F7 and anti-FABP4 mAb 21B8 each bound their recombinant and native antigens with the same apparent affinity (Figure 2B, 2C and Table 1).

### Three other mAbs appeared to distinguish between the recombinant and native forms of their respective antigens

J17 bound purified rhPCSK9 with a twofold higher K_D_ value than serum antigen (Figure 3A) whereas 2B2 bound purified rhPGRN with a twofold lower K_D_ value than serum antigen (Figure 3B). Of the six model interactions that we tested, 33B12 appeared to discriminate most strongly between the recombinant and native forms of its antigen, showing an almost four-fold lower K_D_ value for serum antigen than purified rhFABP4 (Figure 3C).

### Interactions with pH-dependent K_D_ values may appear to have altered affinities in aerated serum samples

**Figure 4:**
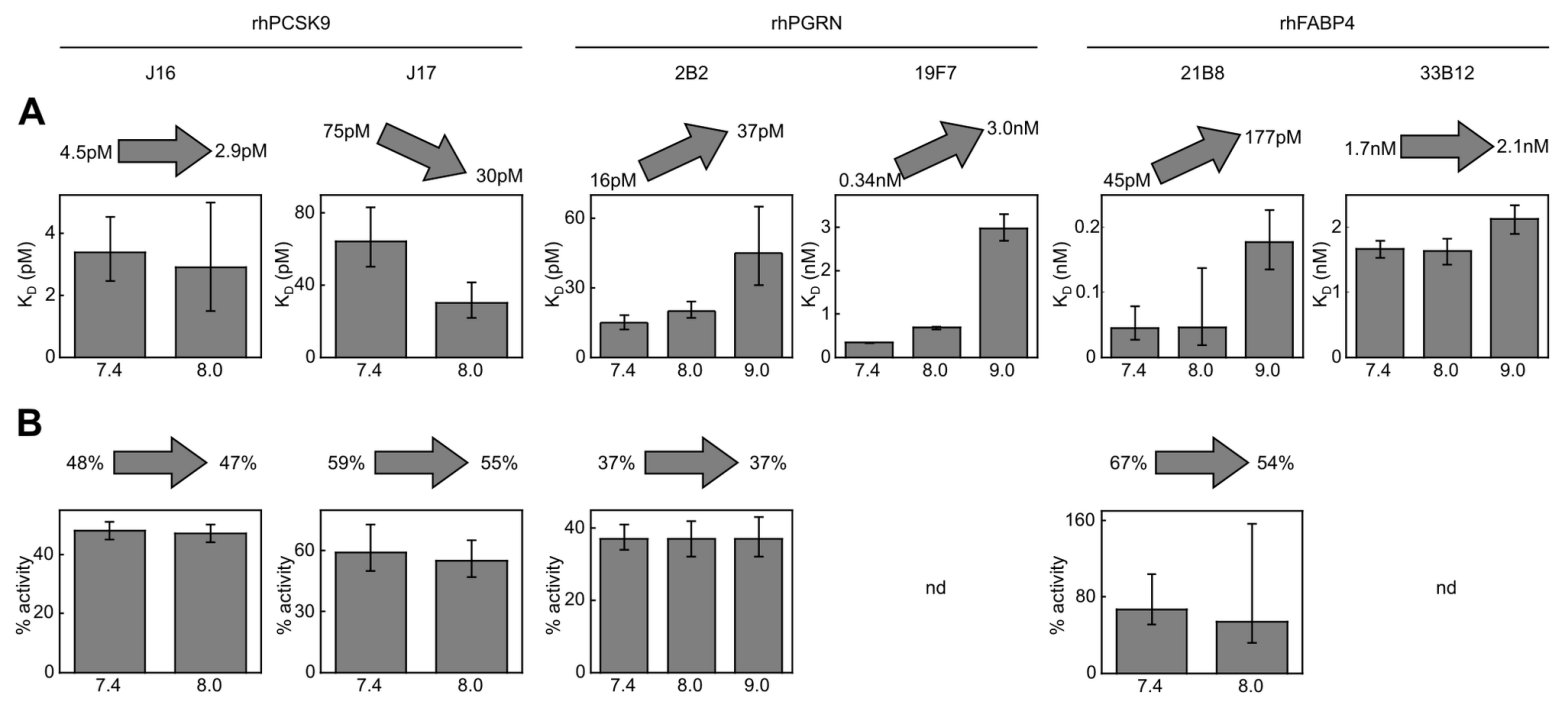
Influence of pH on the apparent affinity (top) and apparent activity (bottom) of different mAbs towards their purified recombinant antigens. The K_D_ values and mAb activities for each interaction were obtained from a single curve KinExA analysis performed at different pH values that spanned the pH range encountered during serum experiments. The bars represent the best fit values and the error bars represent the 95% confidence interval. The arrows indicate the trend observed with increasing pH and the range of best fit values for K_D_ and activity. Only sweet spot experiments enabled a determination of both the K_D_ and the mAb activity (no mAb activity is reported for 19F7 and 33B12 because those curves were mostly K_D_-controlled). The antigen concentrations used were 128 pM rhPCSK9, 42 pM rhPGRN, 21 pM rhPGRN, 100 pM rhFABP4, and 1nM rhFABP4 (from left to right).

It is well established that the pH of aerated serum samples increases over time due to the loss of dissolved carbon dioxide [10]. We therefore investigated whether pH was responsible for the apparent discrepancies that we observed for some mAbs when we compared their affinities towards recombinant and serum antigens. The pH of our undiluted serum samples was consistently above 8.0 upon filtration and preparation of a mAb titration series under aerobic conditions. The pH further increased during incubation (in partially filled capped tubes) and run time (during which the samples were either uncapped or loosely covered with septa caps). We recorded a maximum pH value of 9.2 when undiluted serum samples were allowed to aerate for 2.5 days in open tubes. Serum samples that were diluted in PBS pH 7.4 sample buffer also increased in pH with time and the maximum pH reached in concluding an experiment was dependent on an interplay of factors, notably the dilution factor used and the total time exposed to air, which included the time taken for sample handling, incubation time to equilibrium (which was up to four days for high-affinity interactions such as J16 but only several hours for low-affinity interactions such as 19F7) and run time of the experiment on the KinExA. To investigate whether the affinities of our model interactions were pH-dependent within the pH range encountered during a typical KinExA analysis of serum samples, we studied each mAb’s binding towards its respective recombinant antigen in alkaline buffers (Figure 4). To expedite a pH screen for each studied interaction, we chose an antigen concentration that typically resulted in a “sweet spot” curve (or in some cases a K_D_-controlled curve) and compared the results from a single titration performed in different buffers. We fitted the K_D_ and, where possible, the mAb activity; where the mAb activity could not be determined from a single curve, we used the value obtained from the global fits shown in Figures 2 and 3. While our standard KinExA buffer was based on phosphate buffered saline (PBS, pH 7.4), we used Tris buffered saline (TBS) to obtain more alkaline pH values. To exclude confounding an influence of pH with that of a buffer ion, we confirmed for all interactions that the K_D_ values determined in PBS pH 7.4 were identical to those obtained in TBS pH 7.4 (see Figure S1). 

To span the pH range encountered during the serum experiments shown in Figure 2A, we tested the PCSK9/J16 interaction at pH 7.4 and pH 8.0. Within the 95% confidence limits of each analysis, J16 showed the same apparent affinity and activity at pH 7.4 and pH 8.0 (Figure 4A). For the PCSK9/J17 interaction, however, we observed an approximately two-fold increase in affinity at pH 8.0. The K_D_ value measured for J17 towards rhPCSK9 at pH 8.0 thus matched the value determined for J17 in serum (compare Figures 3A and 4A).

In some of our serum samples titrated with anti-PGRN and anti-FABP4 mAbs, we measured a pH significantly above 8.0. We therefore tested these interactions with recombinant antigens at pH 7.4, 8.0, and 9.0. Increasing the pH to 8.0 and 9.0 resulted in an affinity loss for both anti-PGRN mAbs and the moderate affinity loss determined for 2B2 matched the discrepancy observed between recombinant and serum PGRN. The anti-FABP4 mAb 21B8 showed an affinity loss at elevated pH while 33B12 seemed unaffected. The affinity loss of pH-dependent anti-PGRN and anti-FABP4 interactions was much more pronounced at pH 9.0 than at pH 8.0; however only undiluted serum samples reached a pH of 9.0 in our assays. Although 19F7’s affinity was 10-fold lower at pH 9.0 than pH 7,4, we did not observe any discrepancy in our serum versus recombinant antigen measurements shown in Figure 1B, presumably because the maximum pH recorded for the K_D_-controlled curves in those experiments was barely above pH 7.4 (i.e., pH 7.6 and pH 7.7 for the 1/10 and 1/5 serum dilutions, respectively) due to the relatively short incubation time (a few hours) required to reach equilibrium for this relatively weak affinity binder (K_D_~300pM).

In summary, the subtle (approximately two-fold) differences observed when comparing the interactions with recombinant and serum antigens for J17 and 2B2 likely reflected the influence of pH in these assays, while the four-fold higher affinity of 33B12 for serum FABP4 could not be explained by pH and therefore seemed to indicate a genuine difference between the native and recombinant forms of the antigen.

### For a better comparison between recombinant and serum antigen, the assays should be conducted at the same pH value

**Figure 5:**
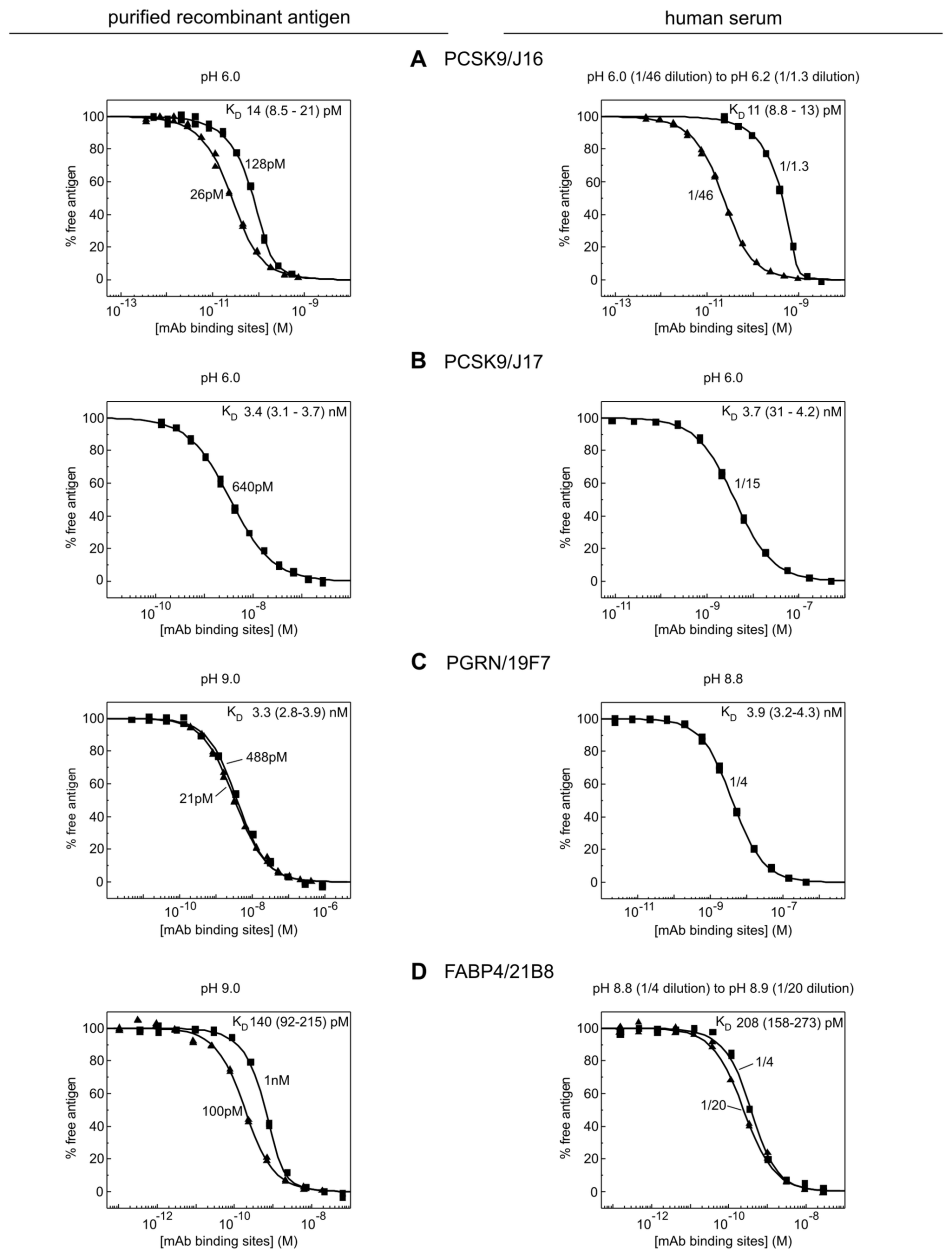
KinExA-determined apparent affinities for four mAbs (J16, J17, 19F7, and 21B8) towards their purified recombinant antigens (left) and their serum antigens (right) at stable, non-neutral pH values. Titration curves are presented as described in Figure 2.

Since the pH drift observed when working with serum under aerobic conditions could confound affinity comparisons of a given mAb towards its purified recombinant antigen and native antigen, we studied select mAbs with recombinant and serum antigens under assay conditions where we matched the pH precisely. We found that serum retained a more stable pH at values of 6.0 (a pH value that is relevant for mAb J16 and its pH-sensitive variant, J17 [9]) and 9.0 (which was close to the highest pH value measured in aerated serum samples in our assays). Figure 5 summarizes the results of these experiments and shows that, for four distinct antigen/mAb interactions, we obtained excellent agreement in the affinities of a given mAb towards its purified recombinant antigen and serum antigen when the two assays were conducted at the same pH value. Furthermore, the results obtained with serum antigens (Figure 5C-D) recapitulated the pH-dependencies observed with recombinant antigens in our “sweet spot” pH scouting experiments (Figure 4A). 

### Apparent mAb activities were not pH-dependent and allowed the determination of serum antigen concentrations

When working with serum, knowing the mAb’s active concentration allows one to determine the concentration of its serum antigen and the K_D_ value of the antigen/mAb interaction. We did not detect any change in any mAb’s apparent activity within the pH range of 7.4 to 9.0 for the interactions tested (Figure 4B), indicating that neither the binding stoichiometry nor the relative concentrations of the two binding partners were affected by alkaline pH. From the data shown in Figure 5A, we also obtained J16’s apparent activity at pH 6.0 which was identical, within the experimental error, to that determined at pH 7.4 (52 (43 - 65)% at pH 6.0 compared to 47 (45 - 51)% at pH 7.4). Therefore, it seemed unlikely that any of our model mAbs or antigens denatured upon exposure to the range of buffer pH values that we explored. However, if both the activity of the antigen and the activity of the antibody change by the same percent while preserving the stoichiometry of the interaction or if a change in stoichiometry exactly compensates a change in activity, this will result in an apparently preserved activity with a possibly altered apparent K_D_ of the interaction. Therefore, we confirmed for rhPGRN that CFCA returned the same apparent activity regardless of the pH of the running buffer (i.e., pH 7.4, 8.0, or 9.0) or the identity of the immobilized anti-PGRN mAb (i.e., 2B2 or 19F7). 

For all three model antigens, the apparent serum concentrations determined by two different mAbs were identical within the 95% confidence interval of the analyses (Table 1) regardless of whether their epitopes were competing - as in the case of anti-PCSK9 mAbs J16 and J17 - or non-competing – as for the other mAbs tested. All antigen concentrations measured in serum also agreed well with literature values [11–15]. It is noteworthy that the apparent PCSK9 concentration varied significantly between serum lots (195 pM for the serum lot shown in Figure 1 and 1 nM for the lot shown in Figures 2, 3, and 5 and Table 1), presumably reflecting the high donor-to-donor variability of PCSK9 serum levels, as reported in the literature [11]. To corroborate the KinExA-determined concentration of PCSK9 for the serum lot used for the experiments in Figures 2, 3, and 5, we also determined its concentration via ELISA. We obtained the same PCSK9 concentration via sandwich ELISA (1.2 nM ± 5% standard deviation) and KinExA (1.1 (0.85 - 1.3) nM) when using the same reagents (mAbs for coating and detection and rhPCSK9 as standard using the CFCA-determined concentration). With a commercially available kit, we obtained a PCSK9 concentration of 5.4 nM (± 4%) when using all provided reagents and 4.8 nM (± 4%) when using our own rhPCSK9 standard.

Since the dilution factor relating the curves within a global fit is incorporated as a fixed parameter in the analysis (in form of the input values for different antigen concentrations in different serum dilutions), only experiments performed with the same lot of serum could be analyzed globally in a KinExA analysis. However, regardless of the serum lot used, the analyses returned the same K_D_ for the PCSK9/J16 interaction (Figures 1 and 2).

### The pH-dependency of PGRN/mAb interactions was corroborated using an SPR-based method

**Figure 6:**
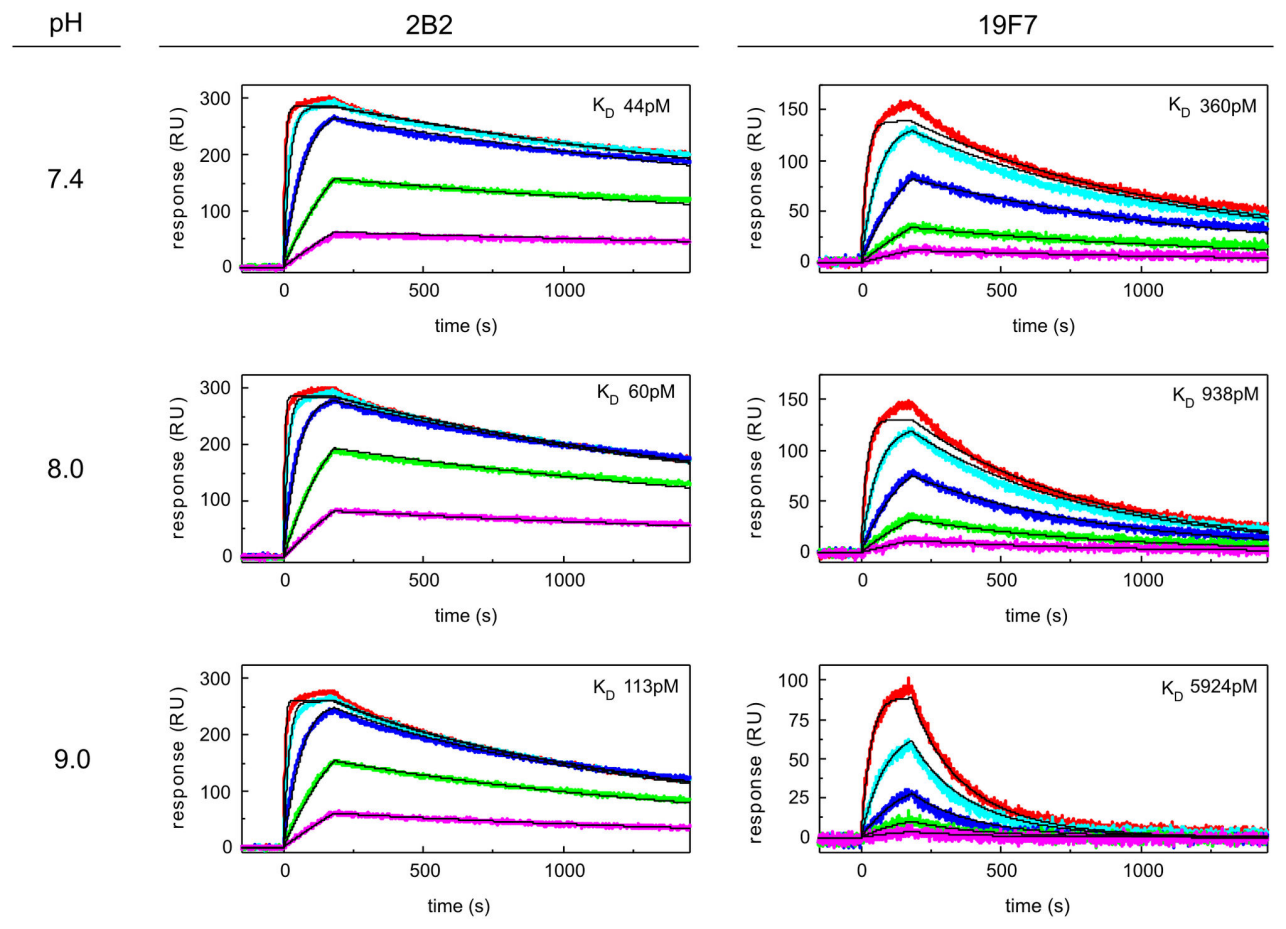
Kinetic analysis of anti-PGRN mAbs 2B2 (left) and 19F7 (right) in TBST buffers at different pH values. The data were collected on a ProteOn XPR36 biosensor by injecting a dilution series of rhPGRN (0.8, 2.4, 7.1, 21.3, and 64 nM) over amine-coupled mAbs. Double-referenced sensorgrams (colored lines) obtained from two ligand channels per mAb were fit globally to a 1:1 binding model with mass transport limitation (fit shown in black); the results from one channel per mAb is shown along with the global best fit K_D_.

We chose one model antigen, namely rhPGRN, to compare the KinExA-determined apparent K_D_ values and their pH-dependencies to those obtained with an orthogonal method. Using the SPR-based ProteOn platform, we performed a one-shot kinetic analysis of rhPGRN in running buffers with pH values of 7.4, 8.0, and 9.0 over amine-coupled 2B2 and 19F7 (Figure 6 and Table 2). For 2B2, the K_D_ value determined in PBST pH 7.4 by SPR (K_D_ = 55 pM) was approximately four-fold higher than that determined via KinExA (K_D_ = 15 pM). For 19F7, the K_D_ values obtained in PBST pH 7.4 via SPR (K_D_ = 382 pM) and KinExA (K_D_ = 335 pM) were identical within the error of the fits. The SPR measurements revealed that both mAbs lost affinity with increasing pH, and that this affinity loss was mostly driven by an increasing dissociation rate constant. Furthermore, the R_max_ values for each mAb were relatively constant over the entire pH range, indicating that the binding stoichiometry of these interactions and the binding capacities (or active concentrations) of the immobilized mAbs were not pH-dependent.

**Table 2:** Kinetic analysis of purified rhPGRN binding to amine-coupled mAbs, as determined via SPR.

**mAb**	**buffer**	**k_a_ (1/Ms)^a^ x 10^6^**	**k_d_ (1/s)^a^ x 10^-4^**	**K_D_ (pM)**
2B2	PBST 7.4	6.13	3.35	55
2B2	TBST 7.4	7.69	3.35	44
2B2	TBST 8.0	7.88	4.71	60
2B2	TBST 9.0	7.41	8.36	113
19F7	PBST 7.4	2.58	9.85	382
19F7	TBST 7.4	2.74	9.87	360
19F7	TBST 8.0	1.68	15.8	938
19F7	TBST 9.0	1.12	66.5	5924

^a^ the standard error for the kinetic parameters in each global fit was ≤ 1%

## Discussion

The binding of mAbs to their native target antigen is an absolute requirement for their therapeutic efficacy. Here, we describe a method to determine the apparent affinities of antigen/mAb interactions to unpurified native antigens contained in human serum samples. All of our model mAbs bound serum antigen even though they were raised via hybridoma technology using the purified recombinant antigen as immunogen. When experiments were conducted in a standard PBS buffer at pH 7.4 with recombinant antigen or in aerobically-handled serum that was either undiluted or diluted into a standard PBS buffer at pH 7.4, some mAbs showed the same apparent affinity for recombinant and native antigens (anti-PCSK9 mAb J16, anti-PGRN mAb 19F7, and anti-FABP4 mAb 21B8), while others showed a slightly different affinity for the native antigen that appeared to be influenced by the alkaline pH of serum samples (anti-PCSK9 mAb J17 and anti-PGRN mAb 2B2). One mAb (anti-FABP4 mAb 33B12) appeared to have a higher affinity for the native antigen than the recombinant antigen. For all interactions that we studied, the experiments conducted in different serum dilutions (including undiluted serum) were well described by a global fit to a 1:1 binding model, suggesting that none of these interactions was perturbed by the highly complex composition of serum, an observation that demonstrates the exquisite binding specificity of mAbs.

The following observations guided our method development:

### The KinExA method is applicable to soluble unpurified antigens in human serum

Using a variety of antigen/mAb interactions, we found that measuring a solution affinity by KinExA of a mAb for its native unpurified antigen, as available in human serum, is not only possible with minimal requirements for sample preparation and assay optimization but can also give remarkably close agreement with affinities obtained towards purified recombinant antigens. To facilitate the assay’s setup, we chose model antigens that were monovalent soluble proteins with easily detectable serum concentrations, of approximately 1 nM. We estimated the serum concentrations of these model antigens by performing a sandwich ELISA with our designated model mAbs. Antigens with lower serum concentrations can be used for a K_D_ determination if the sensitivity of the detection is high enough, which in turn depends upon the availability of mAb pairs employed as bead coat and secondary detection (discussed below). The fixed antigen orientation requires that the antigen’s concentration in a given sample is both detectable and constant during the incubation and assay run time. 

### The alkaline pH of aerobically-handled serum should be taken into account

When working with serum, the fact that its pH increases upon aeration should be taken into account as a possible confounding factor. We did not observe any indication of pH-dependent denaturation for any of our model mAbs or antigens. One way to maintain a relatively stable pH value during a KinExA experiment with serum is to dilute it several fold into the sample buffer, which is typically a standard PBS-based buffer at pH 7.4. High dilution factors (which in turn will lower the working concentration of the serum antigen) will often yield K_D_-controlled curves at a defined pH value, which can be sufficient to determine a K_D_ value. High affinity interactions required long incubation times but a relatively controlled pH could be reached through the use of high dilution factors when permitted by detection sensitivity (e.g., a 50-fold serum dilution titrated with J16 drifted to only pH 7.5 within seven days). While adjusting the serum to pH 7.4 by titration with phosphoric acid was feasible, the pH of concentrated serum could not be maintained properly throughout an entire assay. Therefore, to minimize our sample handling and thus avoid manipulating the serum in unknown ways, we opted for a simple dilution of serum into standard buffers such as phosphate or Tris buffers (with 6 mM to 25 mM buffer ions) with physiological salt concentration (i.e., 150 mM NaCl).

Even though the pH of serum drifted upwards within an assay, we found it useful to obtain an estimate of the serum antigen’s concentration from mostly stoichiometry-controlled curves (representing high serum antigen concentrations) and then analyze the K_D_- and stoichiometry-controlled curves globally to give a robust fit. While the pH often varied more than a unit within such a multi-curve analysis, we did not detect any significant deviation from a simple 1:1 fit which is consistent with our observation that the apparent concentrations of our model antigens were not pH-dependent in the range that we explored (from pH 6.0 to 8.0 for PCSK9 and from pH 7.4 to pH 9.0 for PGRN and FABP4). 

In our assays with undiluted serum, the pH could reach a value of 9.2 when filtered and left uncovered for 2.5 days. (We have observed that conditioned media reaches a similar pH upon aeration – data not shown.) Therefore, we routinely investigated whether the affinities of our interactions were pH-dependent by testing the mAbs against their respective purified recombinant antigens in alkaline buffers. Our pH scouting experiments were facilitated by the use of “sweet spot” curves [3]. Knowing whether the affinity of an interaction is pH-dependent within the alkaline range observed for aerobically handled serum samples allows one to identify a true K_D_ difference between recombinant and serum antigens as opposed to an apparent K_D_ difference that is merely an artifact of conducting the two assays at different pH values. For example, neither the rhPCSK9/J16 nor the rhFABP4/33B12 interaction showed pH-dependent affinities in the pH range of 7.4 - 8.0 or 7.4 - 9.0, respectively (Figure 4A), but J16 appeared to bind rhPCSK9 and serum antigen similarly, whereas 33B12 appeared to bind serum antigen with a higher affinity than rhFABP4. 

### CFCA is a complementary tool for KinExA analysis

While relative affinities of a mAb towards its recombinant and native antigens (i.e., K_D_ ratios) can be determined via KinExA without knowing the mAb’s active concentration, it has to be known accurately if the goal is to determine an absolute K_D_ value of a mAb towards its native antigen in serum and/or the antigen’s concentration in serum. In this context, a mAb’s active concentration may differ from its “nominal” protein concentration as determined by methods such as light absorbance or chromatography which measure total protein content and homogeneity rather than functionally active protein. We have observed that even highly pure mAbs can have low activity towards their specific antigen while mAbs that may be heterogeneous by other methods can retain high activity and homogenous antigen-binding behavior. For example, despite being highly purified, both J16 and J17 were each only about 50% active towards their specific antigen (as noted in Table 1). We therefore aimed to determine the active concentrations of our purified mAbs and recombinant antigens via an independent method that does not rely upon the use of a standard compound, namely Biacore’s CFCA. As explained above (see Results), the CFCA was not amenable to all our reagents but was feasible for all our model antigens. 

### Suitable capture and detection reagents are required

To capture the free antigen on the beads, the titrated mAb itself is often the most convenient choice (as used in our PGRN and FABP4 experiments). If it loses activity upon bead coating or is precious, another mAb that competes with the titrated mAb can be used. In our PCSK9 work, we found that in-house mAb AB1 served as a highly specific bead-adsorbed probe, because it gave excellent signal-to-noise ratios and negligible background signal. AB1-captured PCSK9 paired well with a variety of secondary detection mAbs allowing us to work with the KinExA in a broad linear range between 0.2 V and 7 V total signal amplitude. Since mAbs from the same fusion or panning with a given antigen often fall into multiple epitope bins, a set of possible reagents for bead coating and detection may be readily available. While we preferred a DyLight-coupled sandwiching mAb, to enable a single-step detection, the use of commercial polyclonal antibodies and/or a two-step detection using biotinylated antibodies followed by DyLight-streptavidin is a useful alternative when a sandwiching mAb is not available or does not tolerate excessive DyLight coupling.

## Conclusion

Here, through the complementary use of the KinExA and Biacore’s CFCA, we have demonstrated how to determine both, a mAb’s apparent affinity for its unpurified native antigen and the antigen’s endogenous concentration in human serum. The KinExA thus offers a robust and highly sensitive platform to quantify the binding affinity of a therapeutic mAb towards the native epitope in a physiologically relevant matrix. We also demonstrate that the affinity of some interactions is pH-dependent above pH 7.4, which should be taken into account when working with aerated serum samples that can rapidly increase in pH to values significantly above 8.0. Conducting serum experiments at physiological temperatures and in a CO_2_-controlled environment would provide affinity measurements that are even more relevant to biology. 

## Materials and Methods

### Reagents

Generation and purification of in-house anti-PCSK9 mAbs J16 and J17 has been described elsewhere [8,9]. Murine mAbs raised against rhPGRN and rhFABP4 were generated in-house via conventional hybridoma technology and purified via Protein A. C-terminally 6-His-tagged rhPGRN (residues 18-593) with a predicted molecular weight (MW) of 63 kDa was obtained from R&D Systems (Minneapolis, MN, catalog number 2420-PG). C-terminally 6-His-tagged rhPCSK9 (predicted MW 72 kDa) was expressed and purified as described elsewhere [16]. C-terminally 8-His-tagged rhFABP4 (predicted MW 16 kDa) was purified from transiently transfected HEK 293 cells via Ni-NTA chromatography. Pooled human serum from healthy donors was purchased from Bioreclamation LLC (Westbury, NY). Serum samples were obtained in single-use aliquots stored at -80°C, filtered through a 0.2 µm syringe filter after thawing, and supplemented with 0.02% (w/v) sodium azide; we refer to this preparation as “undiluted serum”. A human PCSK9 ELISA kit was purchased from MBL (Woburn, MA, catalog number CY-8079). When using in-house mAbs for ELISA, one mAb was used for plate coating and a biotinylated sandwiching mAb served as detection reagent followed by high sensitivity streptavidin-HRP (Pierce Biotechnology, Rockford, IL). MAbs were biotinylated using a six-fold molar excess of EZ link NHS-LC-LC-biotin (Pierce Biotechnology) and final absorbance values from duplicate measurements of two different serum dilutions were averaged.

### KinExA experiments

All KinExA experiments were performed at room temperature (approximately 23°C) in the “fixed antigen” orientation on either a KinExA 3000 or a KinExA 3200 instrument with autosampler (Sapidyne Inc, Boise, Idaho) as previously described [3]. Briefly, a constant concentration of antigen was titrated with a mAb, samples were equilibrated at room temperature, and free antigen was detected using customized detection reagents for each antigen-mAb interaction. PCSK9 was captured on polymethylmethacrylate (PMMA) beads coated with mAb AB1 and detected with a DyLight-coupled mouse anti-hPCSK9 mAb. PGRN was captured on PMMA beads that were coated with the same mAb as that used for titration (i.e., 2B2 or 19F7) and detected with a DyLight-coupled version of the other one (i.e., 19F7 or 2B2). FABP4 was captured via 21B8- or 33B12-coated PMMA beads and detected with DyLight-coupled 33B12 or 21B8, respectively. Coupling to DyLight 650 (Pierce Biotechnology) was performed according to the manufacturer’s instructions and all detection mAbs were used at a final concentration of approximately 0.4 µg/mL. The KinExA detection optics consisted of a 620/30 bandpass excitation filter, a 670/40 bandpass emission filter and a 645 nm long pass dichroic mirror. 

If not mentioned otherwise, samples with purified recombinant human antigens were prepared in regular PBS pH 7.4 buffer (1 mM KH_2_PO_4_, 5.6 mM Na_2_HPO_4_, 154 mM NaCl) supplemented with 0.01% (v/v) Tween-20, 0.02% (w/v) sodium azide, and 1 mg/mL BSA (referred to as “PBS sample buffer”). TBS was prepared using 10 mM Tris (pH 7.4, 8.0 or 9.0) and 150 mM NaCl. For KinExA experiments, TBS was supplemented with 0.01% (v/v) Tween-20, 0.02% sodium azide (w/v), and 1 mg/mL BSA. KinExA running buffers always consisted of the buffer type used for sample preparation (at 10 mM) without BSA and azide. The mAb-coated PMMA beads were always diluted into PBS pH 7.4 and the DyLight-coupled mAb was always diluted into PBS sample buffer. 

Unless stated otherwise, regular serum was diluted into PBS sample buffer; acidified serum was diluted into 25 mM sodium phosphate pH 6.0, 150 mM NaCl, 0.01% (v/v) Tween-20, 0.02% (w/v) sodium azide, 1 mg/mL BSA. Serum was acidified by titration with 100 mM H_3_PO_4_ and the added volume was taken into account for the final dilution factor. For serum experiments at pH 9.0, samples were diluted into 25 mM TBS pH 9.0, 0.01% (v/v) Tween-20, 0.02% (w/v) sodium azide, 1 mg/mL BSA. Serum samples for experiments with anti-PGRN mAbs were diluted into 25 mM sodium phosphate pH 7.4, 150 mM NaCl, 0.01% (v/v) Tween-20, 0.02% sodium azide (w/v), 1 mg/mL BSA. 

KinExA data were analyzed using version 3.2.6 of the instrument software and the pH of serum samples was measured using an InLab MicroPro electrode (Mettler Toledo, Columbus, OH).

### CFCA

All CFCA experiments were performed on a Biacore T200 with research-grade CM5 sensor chips at 25°C as previously described [3]. If not mentioned otherwise, either HBST (10 mM HEPES pH 7.4, 150 mM NaCl, 0.05% (v/v) Tween-20) or PBST (PBS pH 7.4, 0.05% (v/v) Tween-20) were used as running buffer. To determine the active concentrations of antigens, mAbs (AB1, 19F7, 2B2, and 21B8) were amine-coupled at 30 µg/mL - 50 µg/mL in 10 mM sodium acetate pH 4.5 or 5.0, typically yielding approximately 10,000 RU. In the same way, anti-idiotype mAbs that blocked the PCSK9/J16 or the PCSK9/J17 interaction were immobilized to determine the active concentrations of J16 and J17. All immobilized mAbs were regenerated either with a 1:200 dilution of phosphoric acid in water or with a 2:1 (v/v) mixture of IgG elution buffer pH 2.8 (Pierce Biotechnology) and 4 M NaCl. 

To determine 2B2’s active concentration, NeutrAvidin (Pierce Biotechnology, catalog number 31000) was immobilized at 100 µg/mL in 10 mM sodium acetate pH 4.5 yielding 12,000 RU. Biotinylated rhPGRN was prepared by incubating rhPGRN with a two-fold molar excess of EZ-link NHS-LC-LC-biotin (Pierce Biotechnology, catalog number 21343) for 30 min at room temperature. After buffer exchange into PBS, biotinylated rhPGRN was captured to a level of 4500 RU on a NeutrAvidin flow cell and regenerated with two 20 sec pulses of 1:200 phosphoric acid between analyte injections.

### Determination of binding kinetics via SPR

The binding kinetics of purified rhPGRN to 2B2 and 19F7 were determined at 25°C on a ProteOn XPR36 biosensor using a GLC sensor chip (Bio-Rad, Hercules, CA). 2B2 and 19F7 were each amine-coupled at two different levels onto the ligand channels by varying the extent of surface activation. Thus, freshly thawed stock solutions of 400 mM EDC and 100 mM SNHS were mixed in a 1:1 v/v ratio in water and diluted to a final dilution factor of 1/500 or 1/600 of each stock solution. Ligand channels were activated with either EDC/SNHS mixture for 3 min, mAbs were coupled for 3 min at 15 µg/mL in 10 mM sodium acetate pH 4.5, and remaining active esters were blocked with 1 M ethanolamine HCl pH 8.5. Final mean immobilization levels were 170 RU and 500 RU for 2B2 and 280 RU and 410 RU for 19F7 with 3% standard deviation along a ligand channel. Purified rhPGRN was injected for 3 min in a “one-shot kinetic” mode at six different concentrations simultaneously along parallel analyte channels (0, 0.8, 2.4, 7.1, 21.2, and 63.6 nM active concentrations, as determined via CFCA). All steps were performed at a flow rate of 30 µL/min and the surface was regenerated with two 18 sec pulses of a 2:1 (v:v) mixture of IgG elution buffer pH 2.8 and 4 M NaCl after each analyte injection. The analysis was performed in various running buffers: PBST pH 7.4; TBST (10 mM Tris, 150 mM NaCl, 0.05% (v/v) Tween-20) pH 7.4, TBST pH 8.0, and TBST pH 9.0. The binding data were double-referenced using the interspots (representing unmodified chip surface) and the in-line buffer blank (“0 nM PGRN” injection). The double-referenced sensorgrams were globally fit within the ProteOn software using a 1:1 binding model with mass transport.

## Supporting Information

Figure S1 **Example of raw data included in the analysis shown in Figure 4.** Titration curves obtained in different buffers are overlaid for (A) 100 pM rhFABP4 titrated with mAb 21B8 and (B) 1 nM rhFABP4 titrated with 33B12.
(TIF)
