# Supplementary figures and images for: Determining the Binding Affinity of Therapeutic Monoclonal Antibodies towards Their Native Unpurified Antigens in Human Serum
Source: PLoS One. 2013 Nov 6;8(11):e80501. doi: 10.1371/journal.pone.0080501 (PMC3819287; doi:10.1371/journal.pone.0080501)

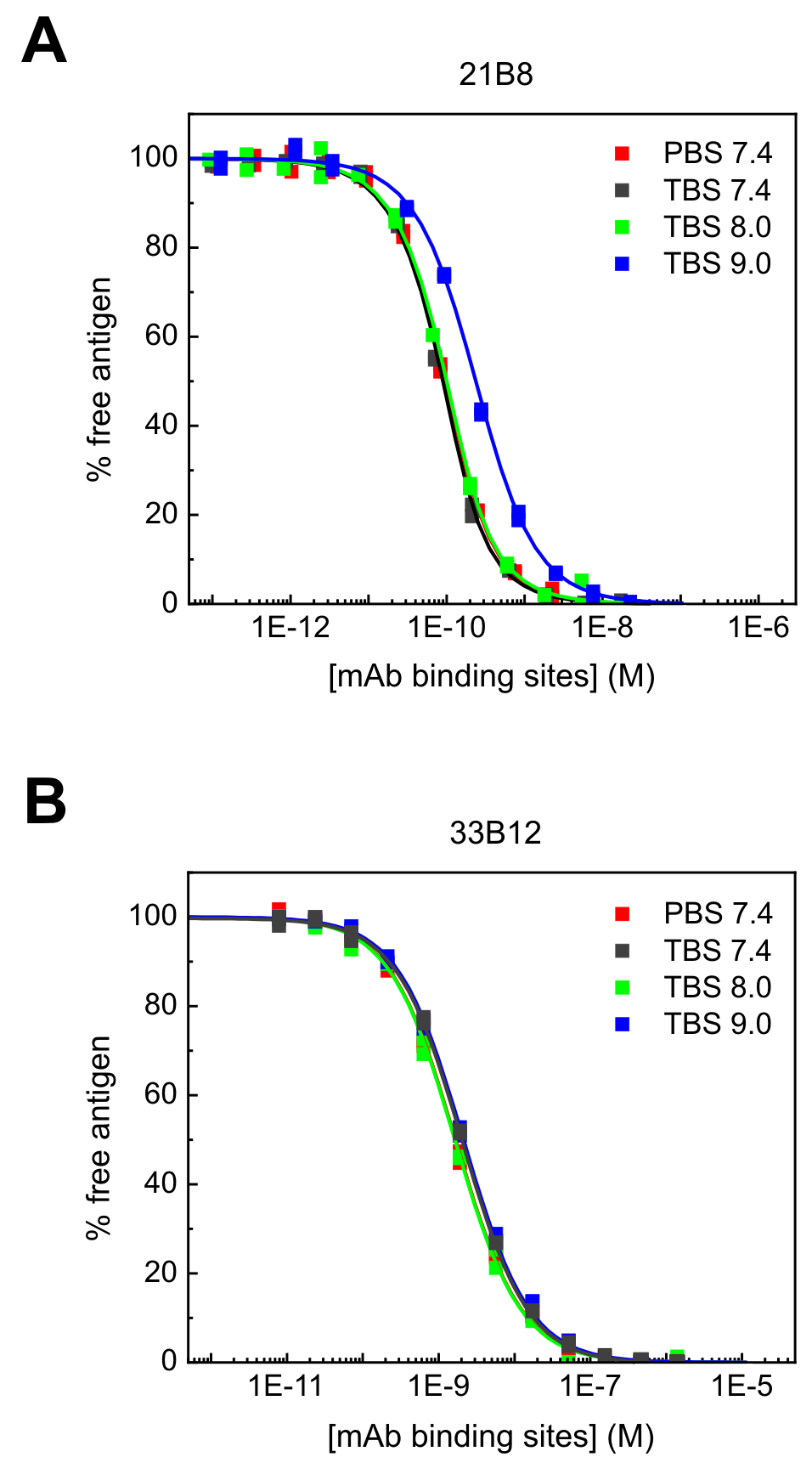

Supplement: Figure S1 — Example of raw data included in the analysis shown in Figure 4. Titration curves obtained in different buffers are overlaid for (A) 100 pM rhFABP4 titrated with mAb 21B8 and (B) 1 nM rhFABP4 titrated with 33B12. (TIF) [file pone.0080501.s001.tif]
